# Identification of Phox2b-regulated genes by expression profiling of cranial motoneuron precursors

**DOI:** 10.1186/1749-8104-3-14

**Published:** 2008-06-19

**Authors:** Patrick Pla, Marie-Rose Hirsch, Stéphane Le Crom, Simone Reiprich, Vincent R Harley, Christo Goridis

**Affiliations:** 1 Ecole normale supérieure, Département de Biologie, 75005 Paris, France; 2 CNRS, UMR8542, 75005 Paris, France; 3 UMR 146 Institut Curie-CNRS, Centre Universitaire, Bat. 110, 91405 Orsay cedex, France; 4 IFR36, Plate-forme Transcriptome, Ecole normale supérieure, 75005 Paris, France; 5 Institut für Biochemie, 91054 Erlangen, Germany; 6 Human Molecular Genetics Laboratory, Prince Henry's Institute of Medical Research, Clayton, Victoria 3168, Australia

## Abstract

**Background:**

Branchiomotor neurons comprise an important class of cranial motor neurons that innervate the branchial-arch-derived muscles of the face, jaw and neck. They arise in the ventralmost progenitor domain of the rhombencephalon characterized by expression of the homeodomain transcription factors Nkx2.2 and Phox2b. Phox2b in particular plays a key role in the specification of branchiomotor neurons. In its absence, generic neuronal differentiation is defective in the progenitor domain and no branchiomotor neurons are produced. Conversely, ectopic expression of Phox2b in spinal regions of the neural tube promotes cell cycle exit and neuronal differentiation and, at the same time, induces genes and an axonal phenotype characteristic for branchiomotor neurons. How Phox2b exerts its pleiotropic functions, both as a proneural gene and a neuronal subtype determinant, has remained unknown.

**Results:**

To gain further insights into the genetic program downstream of Phox2b, we searched for novel Phox2b-regulated genes by cDNA microarray analysis of facial branchiomotor neuron precursors from heterozygous and homozygous *Phox2b *mutant embryos. We selected for functional studies the genes encoding the axonal growth promoter Gap43, the Wnt antagonist Sfrp1 and the transcriptional regulator Sox13, which were not previously suspected to play roles downstream of *Phox2b *and whose expression was affected by *Phox2b *misexpression in the spinal cord. While *Gap43 *did not produce an obvious phenotype when overexpressed in the neural tube, *Sfrp1 *induced the interneuron marker Lhx1,5 and *Sox13 *inhibited neuronal differentiation. We then tested whether *Sfrp1 *and *Sox13*, which are down-regulated by Phox2b in the facial neuron precursors, would antagonize some aspects of *Phox2b *activity. Co-expression of *Sfrp1 *prevented *Phox2b *from repressing Lhx1,5 and alleviated the commissural axonal phenotype. When expressed together with *Sox13*, *Phox2b *was still able to promote cell cycle exit and neuronal differentiation, but the cells failed to relocate to the mantle layer and to extinguish the neural stem cell marker Sox2.

**Conclusion:**

Our results suggest novel roles for *Sfrp1 *and *Sox13 *in neuronal subtype specification and generic neuronal differentiation, respectively, and indicate that down-regulation of *Sfrp1 *and *Sox13 *are essential aspects of the genetic program controlled by Phox2b in cranial motoneurons.

## Background

Branchiomotor (bm) neurons comprise an important class of cranial motoneurons. They arise in the hindbrain and rostral cervical spinal cord from the ventralmost progenitor domain characterized by expression of the transcription factors Nkx2.2, Mash1 and Phox2b [1,2]. Targeted mutation in the mouse has shown that their development crucially depends on *Phox2b *whereas the inactivation of *Nkx2.2 *or *Mash1 *results in only mild defects of bm development [2-5]. In the absence of Phox2b, the differentiation of all bm neurons is arrested at an early stage and their precursors either die by apoptosis or switch fate and become serotonergic neurons. Conversely, Phox2b induces neurons with bm characteristics when misexpressed in the neural tube of chicken embryos [2,6]. Hence, *Phox2b *appears both necessary and sufficient for the implementation of a specific cranial motoneuronal phenotype. Loss and gain of function experiments also show that Phox2b has proneural activity [7] and promotes cell cycle exit and generic neuronal differentiation [8] (see for review [9]). The development of the facial bm (fbm) neurons that arise in rhombomere 4 (r4) has been particularly well studied in the *Phox2b *knockout embryos. A number of downstream target genes have been identified that depend on *Phox2b *for their expression in fbm neurons by examining expression of candidate genes and by a subtractive screening approach. They include generic markers for young post-mitotic neurons (*Tubb3*, *Nfl*, *Ebf2*, *Math3*) and genes more specifically associated with bm neuron differentiation (*Phox2a*, *Islet1*, *Rgs4*, *Tbx20*) ([4,10] and unpublished results).

Despite this wealth of information, only part of the genetic program that depends on *Phox2b *in fbm neurons has been identified. Indeed, the subtractive screening approach proved far from exhaustive since most previously identified downstream genes were not detected and none were found to be up-regulated in the mutants. Here we have used microarrays to compare the patterns of gene expression in fbm precursors from homozygous (*Phox2b*^*LacZ*/*LacZ*^) mutants, in which fbm differentiation is arrested, with that of heterozygous Phox2b (*Phox2b*^*LacZ*/+^) embryos, in which fbm neurons are phenotypically normal. In addition to genes already identified in previous work, a number of new candidates turned up in the screen, among which many were up-regulated in the mutants and might thus be repressed by Phox2b. Most of the down-regulated genes were panneuronally expressed in young post-mitotic neurons. They were thus expected to pop up in the screen, since fewer post-mitotic neurons are produced in the mutants, and were not analyzed further. We selected a few other genes for further study by gain of function experiments in chicken embryos: *Gap43*, whose expression, although panneuronal, was strikingly enhanced in post-mitotic fbm precursors; and two that were up-regulated in the mutants, *Sfrp1 *and *Sox13*. Overexpression of *Sox13*, whose function in neural development has not been studied so far, inhibited neuronal differentiation and the relocation to the mantle layer (ML) caused by *Phox2b *misexpression in the embryonic spinal cord, while that of *Sfrp1 *prevented the repression of the interneuronal marker Lhx1,5.

## Results

### Identification of genes regulated by Phox2b in facial branchiomotor precursors

Phox2b being an important regulator of the development of bm neurons, we wanted to further investigate its function in the specification of bm neurons by searching for novel genes regulated by Phox2b in fbm neuron precursors. We thus chose to compare gene expression in ventral r4 from heterozygous (*Phox2b*^*LacZ*/+^) and homozygous (*Phox2b*^*LacZ*/*LacZ*^) embryonic day 10.5 (E10.5) mutant embryos. At this stage, fbm differentiation is well underway in *Phox2b*^+/+ ^and *Phox2b*^*LacZ*/+ ^animals while no signs of bm differentiation are seen in the homozygous mutants [4]. Four pairs of *Phox2b*^*LacZ*/+ ^and *Phox2b*^*LacZ*/*LacZ *^RNA samples were amplified from microdissected ventral r4, labeled with Cy3 or Cy5, mixed and hybridized to two mouse cDNA microarrays, a microarray of the 15 K NIA probe set representing approximately 12,000 different genes and a home-made NeuroDev microarray representing 2,000 genes chosen for their expression in developing nervous tissue. We adopted as criteria for putative Phox2b-regulated genes a significance analysis of microarray (SAM) score (see Materials and methods) below -3.5 or above 3.5 for genes up- or down-regulated in the mutants, respectively, and a false discovery rate of 5%. By these criteria, 51 genes were found to be down-regulated and 23 up-regulated in the homozygous mutants (Additional files 1 and 2). Surprisingly, only three genes showed a greater than twofold change in expression. However, out of the 12 genes we selected for validation by *in situ *hybridization (ISH), 10 were confirmed as being clearly differentially expressed, despite fold changes as low as 1.14 and 1.13 for two of them (see below). Part of the rather low signal ratios on microarrays may be due to the fact that the floor plate, which should not be affected in the mutants, was always included in the material used for RNA preparation.

**Figure 1:**
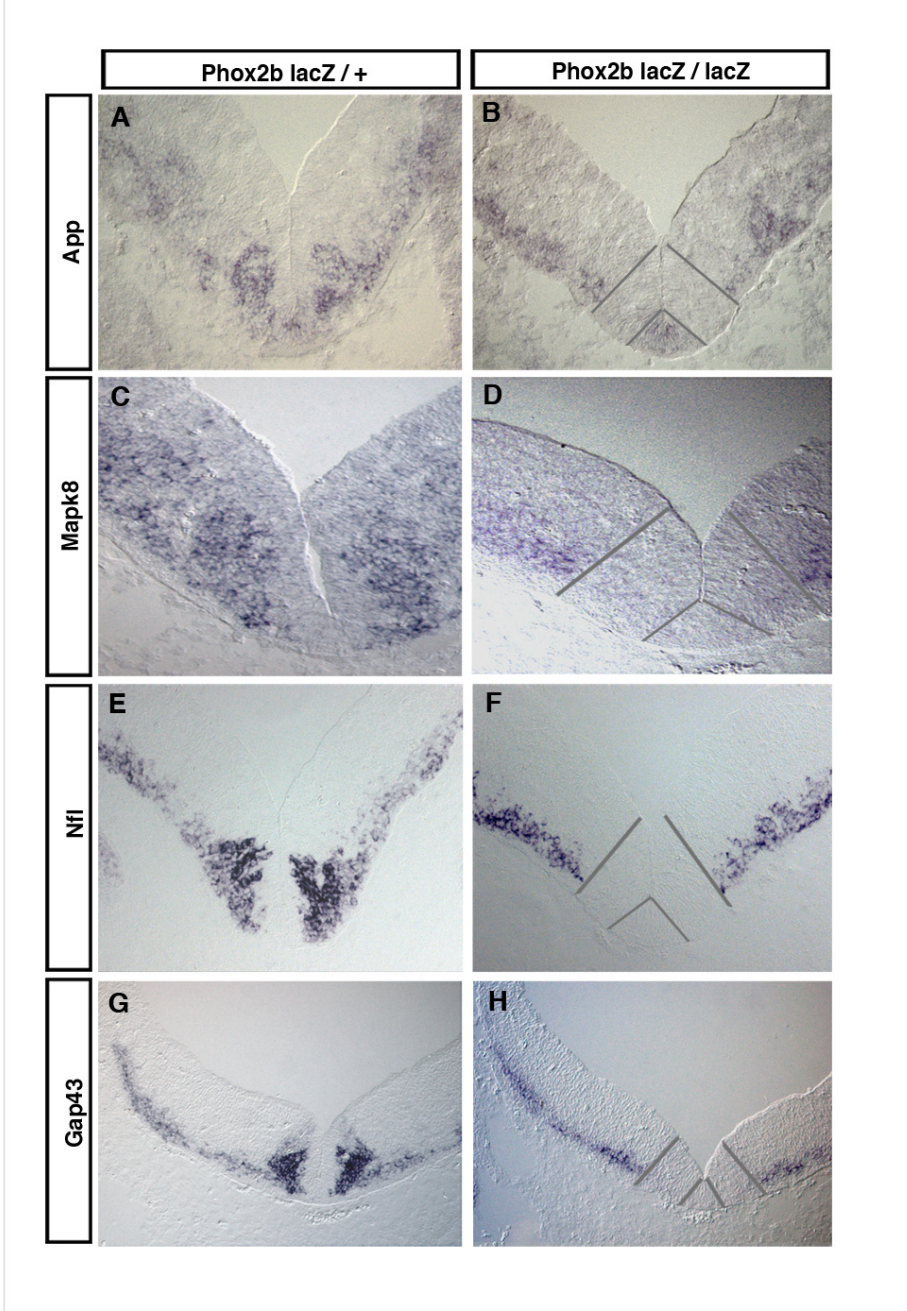
Expression patterns of a subset of genes down-regulated in ventral r4 of *Phox2b*^*LacZ*/*LacZ *^embryos. **(a-h) ***In situ *hybridizations on transverse sections of ventral r4 of *Phox2b*^*LacZ*/+ ^(a, c, e, g) or *Phox2b*^*LacZ*/*LacZ *^(b, d, f, h) E10.5 mouse embryos. DIG-labeled riboprobes recognizing *App *(a, b), *Mapk8 *(c, d), *Nfl *(e, f) and *Gap43 *(g, h) mRNA were used. The normally Phox2b-expressing region as visualized by anti-beta-galactosidase staining on adjacent sections is delimited by gray bars.

Only two of the genes that scored as underexpressed in the mutants, *Tubb3 *and *Nfl*, had been known from previous work ([4] and unpublished data). We selected six for validation by ISH, which was done on sections through r4 from E10.5 *Phox2b*^*LacZ*/+ ^and *Phox2b*^*LacZ*/*LacZ *^embryos. The expression of one of them, *Sncg*, was not noticeably affected in the mutants (not shown). The remaining five fell into two categories. *App*, coding for the amyloid beta (A4) precursor protein, *Mapk8*, also known as Jun N-terminal kinase (*Jnk1*), and *Tubb3*, the *beta3-tubulin *gene (already documented in previous work) are pan-neuronal genes expressed in all developing neurons. In the mutants, their expression was interrupted at the dorsal border of the normally Phox2b^+ ^territory (Figure 1a–d). The small number of post-mitotic neurons that are generated in the homozygous mutants and locate to the ML, some of which are in the process of acquiring a serotonergic fate [1], thus do not appear to activate these genes that are otherwise pan-neuronal. *Gap43*, a major growth cone protein [11,12], and to a lesser extent *Nfl*, coding for the neurofilament light chain, differed from the others by the fact that, although pan-neuronal as well, they were more strongly expressed in the fbm precursors than in other young post-mitotic neurons. Their expression was extinguished in the mutant territory (Figure 1e–h).

**Figure 2:**
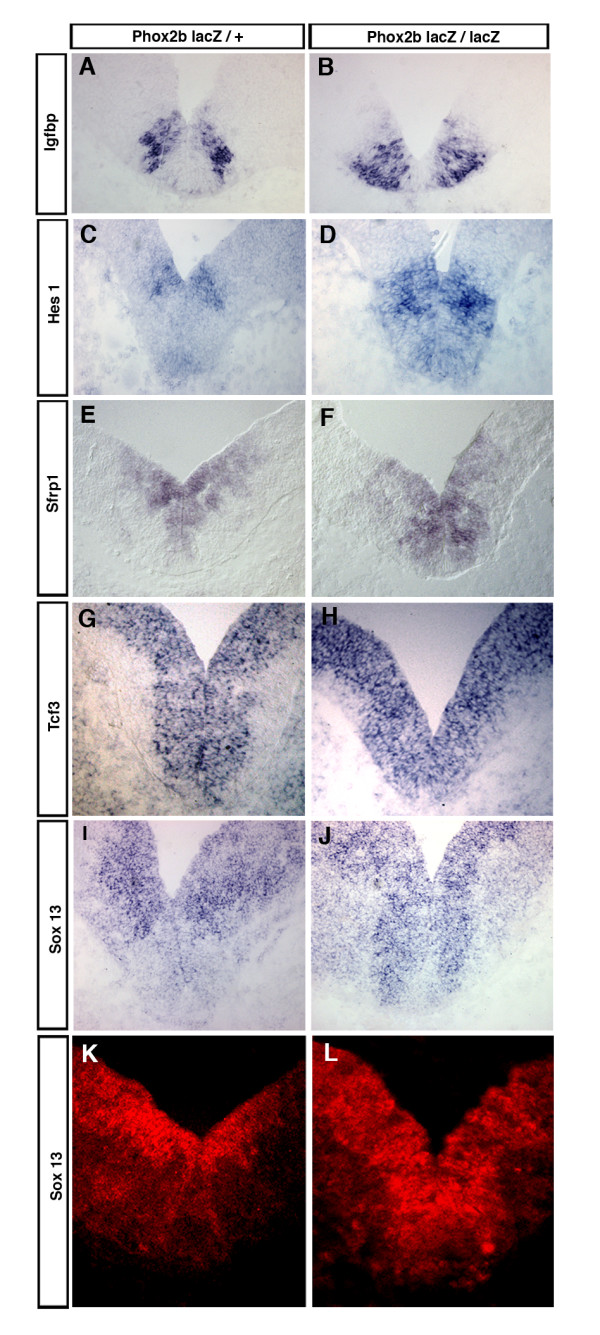
Expression patterns of a subset of genes up-regulated in ventral r4 of *Phox2b*^*LacZ*/*LacZ *^embryos. **(a-j) ***In situ *hybridizations on transverse sections of ventral r4 of *Phox2b*^*LacZ*/+ ^(a, c, e, g, i) or *Phox2b*^*LacZ*/*LacZ *^(b, d, f, h, j) E10.5 mouse embryos. DIG-labeled riboprobes recognizing *Igfbp5 *(a, b), *Hes1 *(c, d), *Sfrp1 *(e, f), *Tcf3 *(g, h) and *Sox13 *(i, j) mRNA were used. **(k, l) **Immunofluorescence with an antibody recognizing Sox13 on transverse sections of ventral r4 of *Phox2b*^*LacZ*/+^(k) or *Phox2b*^*LacZ*/*LacZ *^(l) E10.5 mouse embryos.

In the absence of *Phox2b*, less post-mitotic neurons are generated in ventral r4 and pan-neuronal genes are thus expected to be found down-regulated in the mutants. We therefore favored for further analysis up-regulated over down-regulated genes. We chose six genes among the candidates that were overexpressed in the mutants for validation by ISH. One of them, *Fgfr1*, did not show changes noticeable by ISH (not shown). The others revealed three distinct patterns of changes. In the heterozygotes, *Igfbp5 *was expressed in only part of the *Phox2b*^+ ^territory in the ventricular zone (VZ) and was excluded from the mantle layer (ML), while in the homozygotes, it showed a patchy distribution throughout the *Phox2b*^+ ^VZ and was strongly expressed in the ML (Figure 2a, b). Igfbp5 is a member of a family of modulators of insulin-like growth factor (IGF) activity that may also have IGF-independent functions [13]. Like in our screen, *Igfbp5 *has obtained top scores in other differential screens on neural tissues (see, for instance, [14,15]), signifying perhaps that their expression is exceptionally labile. Their role in neural development has not been explored. Since Igfbp5 has been found to colocalize with areas of apoptosis during development [13], its overexpression may be related to the increased apoptosis caused by lack of *Phox2b *activity in ventral r4. *Hes1*, best known as an effector of Notch signaling [16,17], is normally weakly expressed in the floor plate and more strongly in the dorsalmost part of the *Phox2b*^+ ^VZ in the E10.5 hindbrain, but it invades most of the normally *Phox2b*-expressing VZ in a salt-and-pepper pattern in the mutants (Figure 2c, d).

We found *Sfrp1 *and *Tcf3 *to be normally expressed in and confined to the VZ, *Sfrp1 *only in the ventral part of the basal plate, and *Tcf3 *throughout the neural tube. In the mutant territory, their expression extended up to the neural tube border, thus including the post-mitotic neurons in the ML (Figure 2e–h). A similar expansion into the mutant ML has been found in previous work for *Dll1 *and *Nkx2.2*, which are normally confined to the VZ [4]. In contrast to *Tcf3*, *Sfrp1 *appeared also to be up-regulated throughout the mutant VZ: the signal given by the *Sfrp1 *probe was stronger outside of the fbm progenitors in *Phox2b*^*LacZ*/+ ^embryos, but the reverse was true in *Phox2b*^*LacZ*/*LacZ *^animals. Tcfs are transcription factors that function in the Wnt signaling pathway [18,19]. Secreted frizzled-related proteins (Sfrps) also function mainly in the Wnt pathway as antagonists of Wnt signaling, but Sfrp1 may also exert a Wnt-independent activity as a stimulator of neurite outgrowth and as a growth cone attractant for retinal axons [20-22].

**Figure 3:**
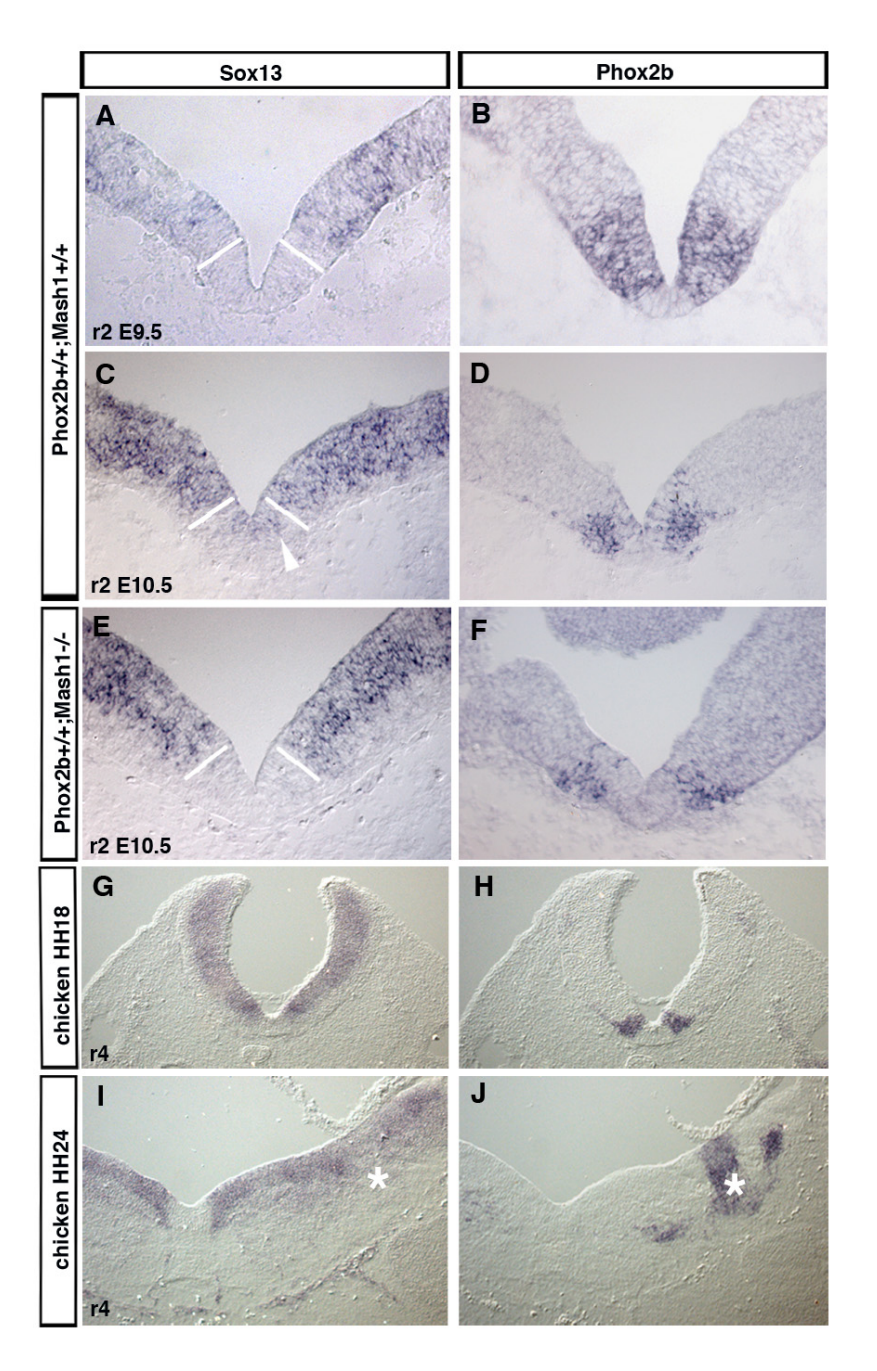
*Sox13 *is down-regulated by *Phox2b*-expressing cells in the hindbrain. **(a-f) ***In situ *hybridizations on transverse sections of ventral r2 from wild-type (a-d) or *Mash1*^-/- ^(e, f) E9.5 (a, b) or E10.5 (c-f) mouse embryos with DIG-labeled riboprobes recognizing *Sox13 *(a, c, e) or *Phox2b *(b, d, f). At E9.5, Sox13 expression is excluded from the ventral progenitor domain expressing *Phox2b*, but invades the progenitor domain when Phox2b expression has disappeared at E10.5 in wild-type (arrowhead), but not in *Mash1*^-/- ^embryos. The *Phox2b*-expressing region as visualized in (b, d, f) is delimited by white bars. **(g-j) ***In situ *hybridizations on transverse sections of ventral r4 of HH18 (g, h) or HH24 (i, j) chicken embryos with DIG-labeled riboprobes recognizing *Sox13 *(g, i) or *Phox2b *(h, j). Asterisks mark a lateral *Phox2b*^+ ^progenitor domain, which does not express Sox13.

The *Sox *genes code for an extended family of SRY-box-containing transcriptional regulators [23]. Sox13, a member of the SoxD group, has been identified as a diabetes autoantigen expressed in pancreatic beta-cells [24] and recently as a positive regulator of gamma-delta T cells [25]. We found *Sox13 *to be expressed throughout most of the VZ, but almost absent in fbm progenitors. In the *Phox2b *mutants, its expression in the normally *Phox2b*^+ ^VZ becomes as strong as elsewhere in the VZ (Figure 2i–l). We then asked whether *Sox13 *would also be excluded from the trigeminal bm progenitors in r2 that also express and depend on *Phox2b*. In ventral r2, *Phox2b *is expressed in the VZ and bm neuron production is underway at E9.5. One day later, bm neuron production has ceased and *Phox2 *is down-regulated in the VZ [7]. At E9.5, strong *Sox13 *expression in the VZ was interrupted at the border of the *Phox2b*^+ ^territory, similar to what has been observed for the E10.5 fbm progenitors. At E10.5, when *Phox2b *expression was about to disappear in the VZ, *Sox13 *became clearly detectable in the ventralmost progenitor domain (Figure 3a–d). Cessation of *Phox2b *expression and bm neuron production in ventral r2 is accompanied by a fate switch of the progenitors, which now produce serotonergic neurons. Their production depends on *Mash1 *activity, in the absence of which serotonergic neurons are not produced [7]. We thus asked whether the expansion of *Sox13 *expression would take place in *Mash1 *mutants. Indeed, in *Mash1*^-/- ^embryos, *Sox13 *expression did not invade the ventral progenitor domain, although *Phox2b *expression has ceased in the VZ (Figure 3e, f).

In Hamburger and Hamilton stage 18 (HH18) chicken hindbrain, we also found *Sox13 *expression very weak or absent in the *Phox2b*^+ ^domain that gives rise to bm/visceromotor neurons, while it extended into the ventral progenitor domain at HH24, when Phox2b has disappeared there. At this stage, a distinct *Phox2b*^+ ^progenitor domain has formed in the basal alar plate. Strikingly, *Sox13 *expression in the VZ was interrupted at this location (Figure 3g–j). Together, these results suggest that Phox2b represses *Sox13 *expression in the hindbrain VZ. The effect may be indirect since *Sox13 *continues to be down-regulated in the progenitors when *Mash1 *is absent.

### *Gap43*, *Sfrp1 *and *Sox13 *are regulated by *Phox2b *misexpression in the spinal cord

We then selected *Gap43*, *Sfrp1 *and *Sox13 *for gain of function experiments in the chicken embryo spinal cord to ask whether they were regulated by ectopically expressed *Phox2b*. We chose *Gap43 *as a panneuronal *Phox2b*-dependent gene because it was more strongly expressed in fbm neurons, *Sfrp1 *as an example of the VZ genes that expand into the mutant ML since it was at the same time up-regulated in the VZ, and *Sox13 *because *Phox2b *repressed it in bm progenitors.

**Figure 4:**
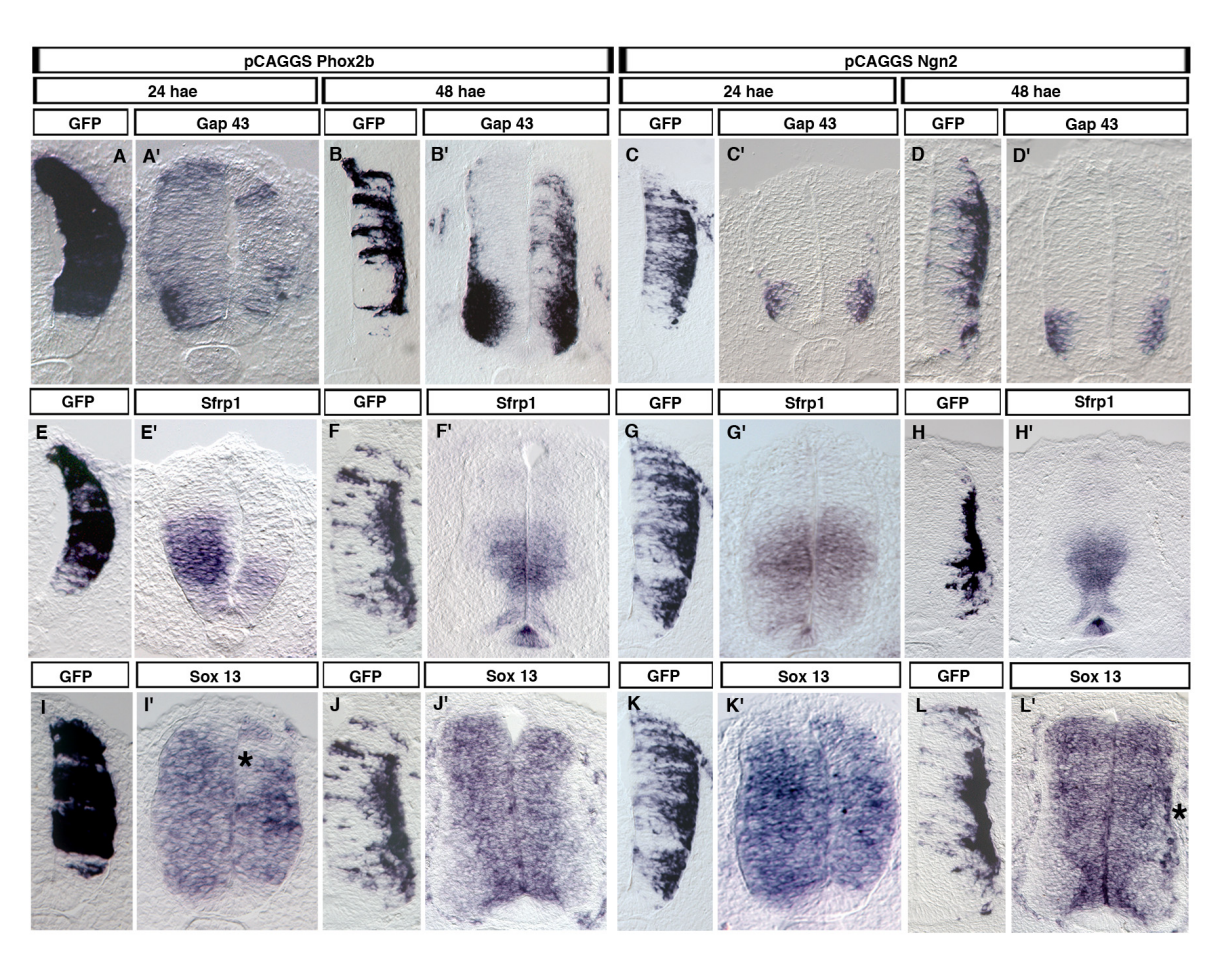
Regulation of *Gap43*, *Sfrp1 *and *Sox13 *expression by *Phox2b *in the chicken spinal cord. **(a-l') **Chicken neural tubes electroporated at HH12-13 with *pCAGGS::Phox2b-IRES-EGFP *(a-b', e-f', i-j') or with *pCAGGS::Ngn2 *together with *pCAGGS::EGFP *(c-d', g-h', k-l') were analyzed 24 or 48 h after electroporation using DIG-labeled riboprobes recognizing *GFP*, *Gap43*, *Sfrp1 *or *Sox13 *mRNA. The asterisk in (i') marks cells in the transfected area where *Sox13 *was clearly down-regulated by *Phox2b*; it was rather up-regulated in adjacent cells. The asterisk in (l') marks cells in the transfected area where *Sox13 *was upregulated by *Ngn2*. Each experiment was repeated three times on different embryos with identical results.

We thus examined whether *Phox2b *electroporated into the spinal cord of HH12-13 chicken embryos could up-regulate *Gap43 *and down-regulate *Sfrp1 *and *Sox13 *expression, in line with the changes observed in *Phox2b *homozygous mutants. *Gap43*, normally expressed at this stage mainly in the post-mitotic motoneurons, was weakly up-regulated in the VZ at 24 hours after electroporation (hae). It was massively induced in the ML at 48 hae (Figure 4a, b). To exclude the possibility that this was a trivial consequence of the fact that Phox2b triggered neuronal differentiation, we overexpressed *Ngn2*, which has a similar propensity to promote neuronal differentiation [6,26]. *Ngn2*, however, had no effect on *Gap43 *expression (Figure 4c, d). *Sfrp1*, normally expressed in a broad band of the VZ, was drastically down-regulated at 24 hae Not surprising for a cell-autonomous effect, *Sfrp1 *expression was only marginally affected at 48 hae, when the *Phox2b*-transfected cells had become post-mitotic and had moved to the ML. *Ngn2 *transfection did not induce changes in *Sfrp1 *expression (Figure 4e–h). The situation with regard to *Sox13 *was more complex. We found *Sox13 *expression to be confined to the VZ. At 24 hae in the *Phox2b*-transfected side, areas where *Sox13 *appeared to be repressed alternated with areas in which *Sox13 *was up-regulated, reflecting perhaps a highly dynamic expression pattern. Such a pattern was not seen after electroporation of *Ngn2*, which had instead a slight propensity of inducing *Sox13 *post-mitotically (Figure 4i–l). These results show that the changes in *Gap43*, *Sfrp1 *and *Sox13 *expression are not a simple consequence of the cells becoming prematurely post-mitotic and suggest that *Phox2b *does not exert its effect through a complex cascade of transcriptional events since the changes are already seen at 24 hae.

### *GAP43 *overexpression does not grossly affect neural tube morphology

**Figure 5:**
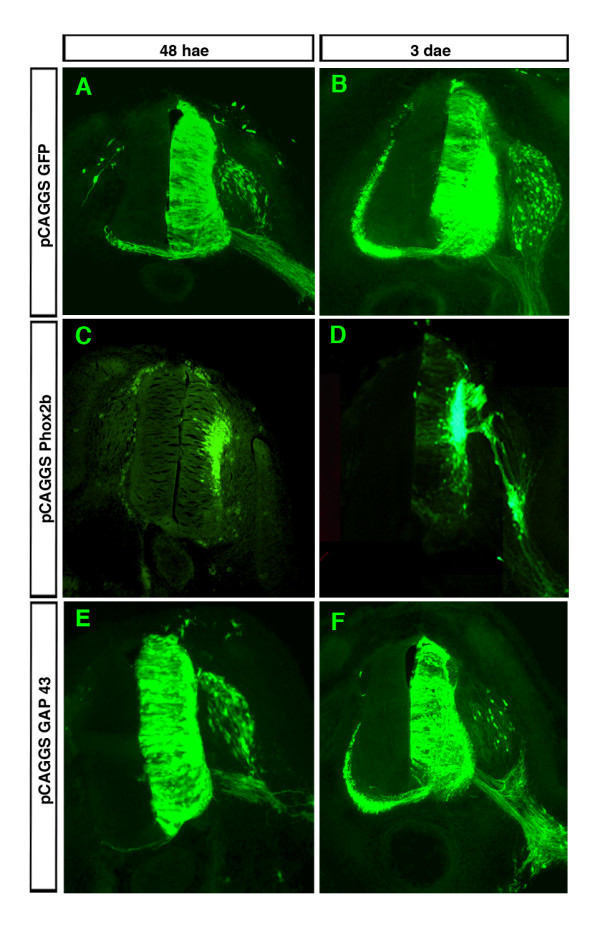
Overexpression of *Gap43 *does not mimic the effects of Phox2b on relocation to the mantel layer and axonal growth. **(a-f) **Vectors expressing either *GFP *alone (a, b) or *Phox2b *and *GFP *(c, d) or *Gap43 *and *GFP *(e, f) were electroporated into the spinal cord of HH12-13 chicken embryos and GFP was visualized by immunohistochemistry 48 hae (a, c, e) or 3 days after electroporation (dae) (b, d, f). Each experiment was repeated three times on different embryos with identical results.

Misexpression of *Phox2b *or its paralogue *Phox2a *in spinal regions of the neural tube induces neuronal differentiation and a bm neuronal fate and imposes an axonal phenotype that shares many features with that of bm neurons. Many dorsally located *Phox2*-transfected neurons, instead of growing commissural axons as they normally do, project into the periphery through dorsal exit points as bm neurons do, but also at ectopic sites [2] (Figure 5c, d). As *Gap43 *is up-regulated by Phox2b both in fbm precursors and when misexpressed in the spinal cord, we thus tested whether it would mimic this phenotype and, in particular, induce axonal growth when overexpressed in the chicken neural tube. However, after electroporation of *Gap43*, the morphology of the neuroepithelium remained grossly normal. Neither did the cells relocate to the ML nor were there any signs of aberrant or increased axonal growth as produced by *Phox2b *(Figure 5e, f).

### *Sfrp1 *and *Sox13 *overexpression counteracts some of the effects of *Phox2b *misexpression

**Figure 6:**
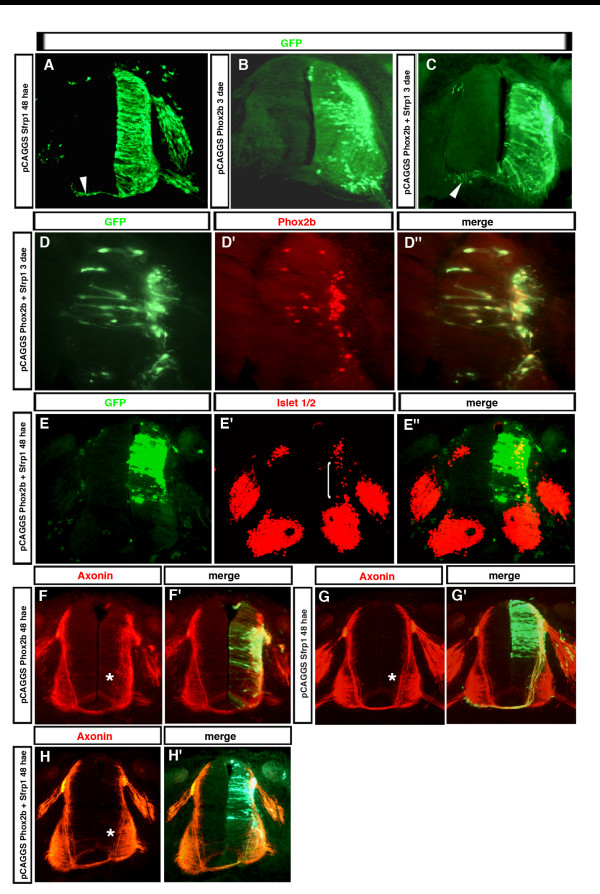
*Sfrp1 *partially restores a commissural axonal phenotype. **(a-c) **Chicken neural tubes electroporated at HH12-13 with *pCAGGS::Sfrp1-IRES-EGFP *(a), *pCAGGS::Phox2b-IRES-EGFP *(b) or *pCAGGS::Sfrp1-IRES-EGFP *plus *pCAGGS::Phox2b-IRES-EGFP *(c) were analyzed by anti-GFP immunohistochemistry. Expression of *Sfrp1 *did not grossly alter the morphology of neuroepithelial cells, neither did it prevent *Phox2b *from promoting relocation to the ML. However, *Sfrp1 *prevented *Phox2b *from repressing the growth of commissural axons (arrowheads). **(d-d") **Chicken neural tubes electroporated with *pCAGGS::Sfrp1-IRES-EGFP *and a *Phox2b *expression vector were analyzed by anti-GFP and anti-Phox2b immunohistochemistry as indicated. Virtually all the transfected cells express both GFP and Phox2b. **(e-e") **Chicken neural tubes electroporated with *pCAGGS::Sfrp1-IRES-EGFP *plus *pCAGGS::Phox2b-IRES-EGFP *were analyzed by anti-GFP and anti-Islet1,2 immunohistochemistry as indicated. Co-expression of *Sfrp1 *did not prevent *Phox2b *from inducing Islet1,2 (bracket). (**f-h') **Chicken neural tubes electroporated with *pCAGGS::Phox2b-IRES-EGFP *(f, f'), *pCAGGS::Sfrp1-IRES-EGFP *(g, g') or with *pCAGGS::Sfrp1-IRES-EGFP *plus *pCAGGS::Phox2b-IRES-EGFP *(h, h') were analyzed by anti-axonin-1 and anti-GFP immunohistochemistry. In (f, g, h), the anti-axonin-1 staining is shown alone, and in (f', g', h') it is merged with the anti-GFP immunofluorescence. The fascicle formed by the commissural fibers *en route *to the floor plate is marked by an asterisk; it is absent after *Phox2b *transfection and partially restored by co-expressing *Sfrp1*.

Overexpression of *Sfrp1 *in the chicken embryo spinal cord also did not produce gross anatomical alterations in the neural tube detectable by green fluorescent protein (GFP) labeling (Figure 6a, compare with Figure 5a). Since *Sfrp1 *appeared to be repressed by *Phox2b *in fbm precursors and when misexpressed in the spinal cord, we asked whether *Sfrp1 *would counteract some of the effects produced by *Phox2b *misexpression. After co-electroporation of *Sfrp1 *together with *Phox2b*, virtually all transfected cells expressed both GFP from the *Sfrp1 *expression vector and Phox2b, showing that co-expression is highly efficient (Figure 6d). When expressed together with *Phox2b *in this way, *Sfrp1 *did not prevent *Phox2b *from either driving neuronal differentiation and relocation to the ML or promoting ectopic neural tube exit of axons (Figure 6c, d; data not shown).

However, forced expression of the *Phox2 *genes [2] (Figures 5c and 6b) suppresses the growth of commissural axons, while cells co-transfected with *Sfrp1 *and *Phox2b *clearly grew commissural fibers as revealed by GFP staining in five out of five electroporated embryos (that is, n = 5/5; Figure 6c). To visualize the trajectory of pre-crossing commissural axons in electroporated embryos, we used anti-axonin-1 (TAG-1 in mammals) antibodies known to strongly label commissural fibers [27]. *Sfrp1 *overexpression did not change the normal pattern of extension of commissural axons, which form a conspicuous fascicle running along the medial border of the motoneuron column (Figure 6g; n = 4/4). As expected, the axonin-1-expressing fascicle was absent at the transfected side after *Phox2b *electroporation (Figure 6f; n = 9/9). Still, anti-axonin-1-labeled fibers were observed in the *Phox2b*-transfected dorsal spinal cord, but they neither formed a tight fascicle nor extended beyond the dorsal border of the motoneuron column. Co-expressing *Sfrp1 *together with *Phox2b *partially, but not completely, restored the axonin-1-labeled fascicle that extends towards the floor plate (Figure 6h; n = 6/6).

**Figure 7:**
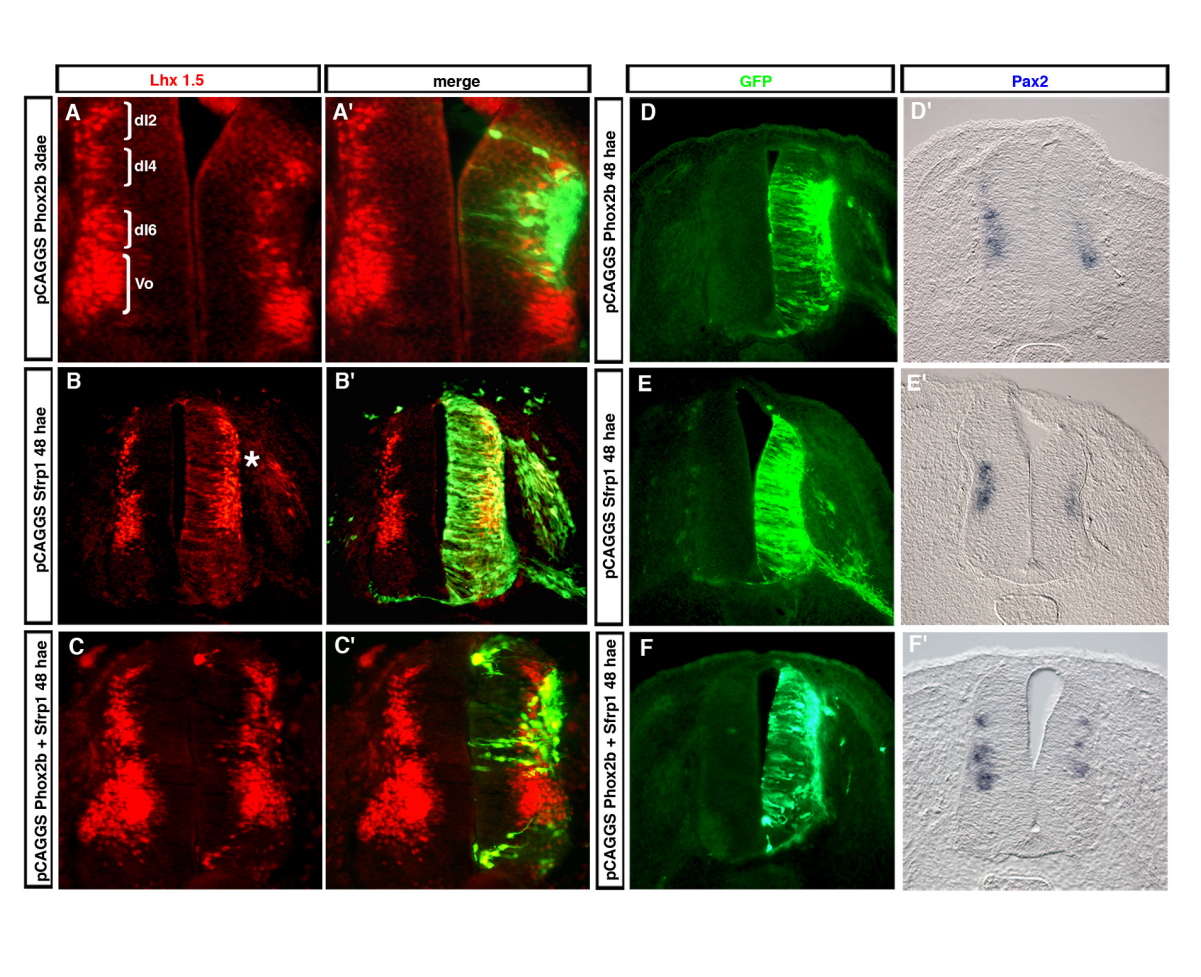
Changes in Lhx1,5 and *Pax2 *expression after *Sfrp1 *transfection. **(a-c') **Chicken neural tubes electroporated at HH12-13 with *pCAGGS::Phox2b-IRES-EGFP *(a, a'), *pCAGGS::Sfrp1-IRES-EGFP *(b, b') or with *pCAGGS::Sfrp1-IRES-EGFP *plus *pCAGGS::Phox2b-IRES-EGFP *(c, c') were analyzed by anti-Lhx1,5 and anti-GFP immunohistochemistry. In (a, b, c), the anti-Lhx1,5 staining is shown alone, and in (a', b', c') it is merged with the anti-GFP immunofluorescence. *Phox2b *represses Lhx1,5 normally expressed in interneuron populations dI2, dI4, dI6 and V0 (brackets), while Sfrp1 increases the number of Lhx1,5^+ ^cells (asterisk) and counteracts *Phox2b *in its capacity of repressing Lhx1,5 (compare (a) and (c)). **(d-f') **Chicken neural tubes electroporated at HH12-13 with *pCAGGS::Phox2b-IRES-EGFP *(d, d'), *pCAGGS::Sfrp1-IRES-EGFP *(e, e') or with *pCAGGS::Sfrp1-IRES-EGFP *plus *pCAGGS::Phox2b-IRES-EGFP *(f, f') were analyzed by anti-GFP immunohistochemistry or by ISH with a *Pax2 *probe as indicated. *Pax2 *is down-regulated in all three conditions.

The commissural axonal phenotype prompted us to ask whether *Sfrp1 *may counteract some of the fate changes affected by Phox2b. Sustained expression of *Sfrp1 *together with *Phox2b *reduced, but did not abrogate, the induction of Islet1,2 (Figure 6e; n = 3/3), normally seen after transfecting Phox2b alone [2,6]. *Phox2b*, in addition to inducing bm markers, represses the interneuronal marker Lhx1,5 [2] (Figure 7a), which is normally expressed by the interneuron populations dI2, dI4, dI6 and V0 [28,29]. This effect was offset by co-expressing *Sfrp1 *(Figure 7c; n = 5/5). Hence, the cells now expressed a mixed phenotype, which is not normally encountered: *Phox2b*^+^, *Islet1*, *2*^+^, Lhx1,5^+^. *Sfrp1 *increased the number of Lhx1,5-expressing cells in the dorsal spinal cord also when expressed alone, explaining its ability to counteract the down-regulation of Lhx1,5 caused by *Phox2b*. After *Sfrp1 *electroporation, the gap in Lhx1,5 expression corresponding to the dI5 neurons was not observed anymore at the transfected side (Figure 7b; n = 9/10). A fainter but clearly visible signal was also seen in the *Sfrp1*-transfected VZ in cells with neuroepithelial morphology, whereas it was never observed in control conditions, suggesting that Lhx1,5 is induced ectopically in the presence of *Sfrp1 *(Figure 7b; n = 9/10). Finally, we examined *Pax2*, which is expressed by several Lhx1,5^+ ^populations [28], as a second marker of spinal interneurons. As expected, misexpression of *Phox2b *also down-regulated *Pax2 *in the transfected cells (Figure 7d; n = 3/3). In contrast to Lhx1,5, however, *Pax2 *was down-regulated as well by overexpressing *Sfrp1 *alone (Figure 7e; n = 3/3), and consequently, *Sfrp1 *did not prevent *Phox2b *from down-regulating *Pax2 *(Figure 7f; n = 3/3). Hence, *Sfrp1 *overexpression affects the phenotype of the neurons that are generated and thwarts the ability of *Phox2b *to induce a bm phenotype.

*Sfrp1 *(*SARP2*) has been found to sensitize cells in culture to pro-apoptotic stimuli, whereas *Sfrp2 *had the opposite effect [30] and prevented programmed cell death in the neural crest [31]. We thus tested whether *Sfrp1 *might influence cell death in our overexpression paradigm. In line with published data [32], we found very few apoptotic cells in the neural tube at the stages studied (one to four TUNEL^+ ^cells per section), and their number differed neither between electroporated and non-electroporated sides of the neural tube nor among different conditions (electroporation of *Srp1 *or *Phox2b *or of *Sfrp1 *plus *Phox2b*) (not shown).

**Figure 8:**
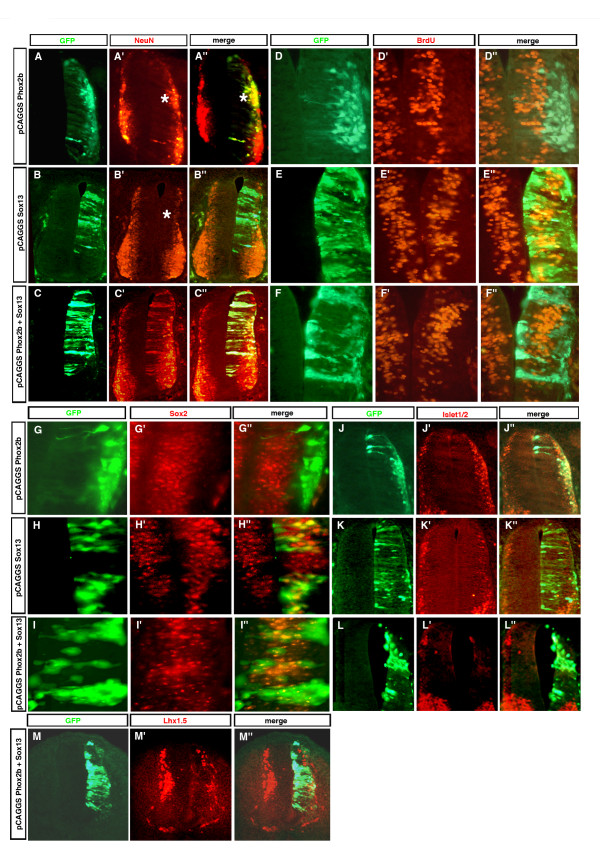
*Sox13 *overexpression partly antagonizes Phox2b activity. **(a-m") **Chicken neural tubes electroporated at HH 12–13 either separately with *pCAGGS::Phox2b-IRES-EGFP *(a-a", d-d", g-g", j-j") or *pCAGGS::Sox13-IRES-EGFP *(b-b", e-e", h-h", k-k") or with *pCAGGS::Phox2b-IRES-EGFP *plus *pCAGGS::Sox13-IRES-EGFP *(c-c", f-f", i-i", l-l", m-m") were analyzed 48 h after electroporation. Transverse sections were stained with anti-GFP antibodies in combination with anti-NeuN (a-c"), anti-Sox2 (g-i"), anti-Islet1/2 (j-l") or anti-Lhx1,5 immunohistochemistry (m-m") or in combination with anti-BrdU antibodies (d-f") after BrdU injection into the amniotic cavity 2 h before fixation. *Phox2b *induces and *Sox13 *represses NeuN (asterisks in (a', a", b')) in the ML. When co-expressed with *Sox13*, Phox2b is still capable of inducing NeuN, which, however, is now also switched on in the VZ (c', c"). Cells electroporated with *Phox2b *are BrdU-negative whether *Sox13 *was co-transfected (f) or not (d). Cells electroporated with *Sox13 *whether co-transfected with *Phox2b *(i) or not (h) express Sox2, while cells electroporated with *Phox2b *alone are always Sox2-negative (g). Islet1,2 induction by *Phox2b *(j) is abolished by co-transfection of *Sox13 *(l). Repression of Lhx1,5 by Phox2b is not prevented by co-expressing Sox13 (m). Co-transfection of *Sox13 *together with *Phox2b *inhibits relocation to the ML (c, f, i, l, m) implemented by expression of *Phox2b *alone (a, d, g, j).

In the chicken and mouse VZ, cells highly expressing *Sox13 *alternate with cells that express it less (Figures 2i, k and 4i', j'), a pattern that seems to be accentuated by overexpressing *Phox2b *(Figure 4i'). Such a pattern suggests that *Sox13 *expression in neuroepithelial cells is dynamic, perhaps in relation to their commitment to a neuronal fate. VZ expression of *Sox13 *driven by a strong constitutive promoter should cancel out these changes in expression. When overexpressed in this way, *Sox13 *did not disrupt neural tube structure or grossly alter neuroepithelial cell morphology. The transfected cells incorporated bromo-deoxyuridine (BrdU; Figure 8e; n = 3/3) and most expressed the VZ cell marker Sox2 (Figure 8h; n = 3/3). However, there were less cells expressing the neuronal marker NeuN in the transfected area, and none of the transfected cells co-expressed NeuN (Figure 8b; n = 3/3), indicating that constitutively high expression of *Sox13 *inhibits neuronal differentiation.

We next tested the idea that *Sox13 *overexpression may antagonize *Phox2b *in its capacity to drive neuronal differentiation. *Phox2b *misexpressed alone causes the transfected cells to accumulate in the ML and to express NeuN (Figure 8a), as expected from its propensity to promote neuronal differentiation and relocation to the ML. When *Sox13 *was misexpressed together with *Phox2b*, many transfected cells remained in the VZ where they either retained the morphology of neuroepithelial cells or aggregated near the lumen of the neural tube (Figure 8c, f, l; n = 3/3). *Phox2b *was still able to induce NeuN, but now many cells double-positive for NeuN and GFP were located in the VZ and had the morphology of neuroepithelial cells. Hence, *Sox13*, although unable to inhibit induction of NeuN by Phox2b, appears to prevent the transfected cells from losing their neuroepithelial morphology and from relocating to the ML. We then investigated further the phenotype of the cells constitutively co-expressing *Phox2b *and *Sox13*. We first established that, in the presence of *Sox13*, *Phox2b *was still able to promote cell cycle exit as shown by the fact that the transfected cells did not incorporate BrdU (Figure 8f; n = 3/3). However, while the neural stem cell marker Sox2 [33] was never expressed in cells transfected with *Phox2b *alone, many doubly transfected cells still expressed Sox2 (Figure 8g, i; n = 3/3). Finally, we examined Islet1,2 as a marker for the bm phenotype induced by *Phox2b*, and Lhx1,5 as a marker for interneurons. Induction of Islet1,2 was completely repressed by co-transfection with *Sox13 *(Figure 8j, l; n = 3/3). By contrast, *Sox13 *did not prevent down-regulation of Lhx1,5 after *Phox2b *transfection, a result to be expected since Sox13 overexpression inhibits neuronal differentiation (Figure 8m; n = 3/3).

## Discussion

Phox2b plays a key role in the formation of two important classes of cranial motoneurons, the bm and the closely related visceromotor neurons. Its role in motoneuronal differentiation has been studied in most detail during differentiation of the fbm neurons. This work has yielded a few genetic targets whose expression is dependent on *Phox2b *in fbm precursors [4,10]. To search for Phox2b-regulated genes by a more systematic approach, we have used gene expression profiling of ventral r4, in which the fbm precursors arise, from heterozygous and homozygous *Phox2b *mutant embryos. In this way we have identified a number of candidates that are either up- or down-regulated in the homozygous mutants. Two of these candidates regulated by Phox2b had been identified in previous work, but none of the others have been reported before. We restrict our discussion to those candidates we confirmed by ISH as being regulated by Phox2b in ventral r4.

Our analysis did not distinguish between genes that were under direct control of Phox2b and genes that depended on Phox2b indirectly, through the action of other transcription factors. However, the genes that we found induced or repressed in the chicken spinal cord already at 24 hae can be supposed to be proximal targets in the genetic program induced by Phox2b, and not to be dependent on a vast network of other factors. So far, only transcriptional activation by Phox2b has been demonstrated, but the possibility exists that it may also act as a repressor, as indicated by the observation that repressor forms of Phox2b reproduced some of the effects of Phox2b [6].

Since less post-mitotic neurons are produced in the mutant territory, it was to be expected that many genes that are normally expressed in young post-mitotic neurons would be underrepresented in *Phox2b*^*LacZ*/*LacZ *^embryos. *App*, *Mapk8*, *Nfl *and *Gap43 *belong to this category. Although post-mitotic cells are produced by *Phox2b*^*LacZ*/*LacZ *^progenitors and migrate to the ML, albeit in reduced numbers, the ML cells failed to express these genes. The same pattern has been found in previous work for other post-mitotic markers [4]. Some of the post-mitotic cells that arise in the mutants then go on to become serotonergic neurons [1,7]. Apparently, in the absence of *Phox2b *and its proneural activity, these cells will acquire neuronal properties only at later stages. *Gap43 *is a case apart since its expression in post-mitotic neurons was distinctly stronger in fbm precursors than elsewhere in r4, suggesting that high *Gap43 *activity may be required for proper fbm differentiation. *Gap43 *was also markedly up-regulated by ectopic *Phox2b *expression in the spinal cord, an effect that could not be ascribed to an increase in generic neuronal differentiation since *Ngn2 *overexpression was without effect. Gap43 was originally described as a protein associated with growing axons, both in development and during regeneration, and is thought to function as a determinant of axonal growth competence [11,12,34]. Despite a wealth of *in vitro *data describing its growth-promoting properties, its *in vivo *function is still ill-defined. When constitutively expressed in adult mice, GAP43 induced branching of motor nerve terminals and promoted sprouting of nerves after lesion [35]. In our study, *Gap43 *overexpression in the embryonic neural tube did not produce overt morphological alterations. Hence, its effects might be limited to terminal fields and situations of regeneration. A number of discrete developmental defects have been reported in *Gap43 *mutants, including pathfinding defects of the optic nerve [36], a reduction in serotonergic innervation of the forebrain [37] and a failure to form the anterior commissure [38]. Such a subtle phenotype for a gene expressed in virtually all young neurons may be due to redundancy with the *CAP23 *gene [39].

Among the genes found to be up-regulated in mutant ventral r4, *Hes1*, at the stage studied, was expressed only in the dorsalmost part of the Phox2b^+ ^VZ in wild-type or heterozygous animals. In *Phox2b*^*LacZ*/*LacZ *^embryos, its expression invaded most of the normally Phox2b^+ ^domain. The basic-helix-loop-helix transcription factor of the *hairy and enhancer of split *family, Hes1 is best known as an effector of Notch signaling and, together with its paralogues Hes3 and Hes5, plays a crucial role in maintaining the progenitor state of neuroepithelial cells [16,17]. Since Phox2b drives neural progenitors to exit the cell cycle and to differentiate, it is perhaps not surprising that it shuts off a negative regulator of neural differentiation. This scheme, however, is complicated by the observation that its paralogue *Hes5 *has been found to be down-regulated in *Phox2b*^*LacZ*/*LacZ *^embryos [8]. It appears that the lack of proneural activity in *Phox2b *mutants and the consecutive down-regulation of the Notch ligand Dll1 in the VZ affect differently the two paralogous genes. The Lef/Tcf proteins are transcription factors that play pivotal roles in Wnt signaling, functioning mainly as repressors in the absence and as obligatory co-activators of beta-catenin in the presence of Wnt ligands [19]. Tcf3 has remained the most enigmatic member of the family. Loss of *Tcf3 *results in early gastrulation defects and perhaps as a consequence to broad defects in neural tube patterning [40]. *Tcf3 *has been said to become undetectable in the mouse embryo from E10.5 onward [18], but we found it strongly expressed in E10.5 r4 throughout the VZ. Normally excluded from the ML, its expression domain invaded the ML in the mutant territory. This has been observed in previous work also for *Nkx2.2 *and *Dll1 *[41]. Apparently, the post-mitotic cells that accumulate in the mutant ML are not only unable to express generic neuronal markers but also do not switch off genes whose expression is normally confined to the VZ.

*Sfrp1 *shared with *Tcf3 *the tendency to be expressed in the mutant ML, from which it was normally excluded. However, the expression pattern of *Sfrp1 *in r4 was much more circumscribed than that of *Tcf3*. It was only expressed in ventral r4, including the *Phox2b*^+ ^progenitor domain and extending up to the middle of the basal plate. In *Phox2b*^*LacZ*/+ ^embryos, its expression was stronger dorsal to the *Phox2b*^+ ^progenitors, but the reverse was true in *Phox2b*^*LacZ*/*LacZ *^embryos. In the embryonic chicken spinal cord, we found *Sfrp1 *to be strongly expressed in the VZ of the dorsal basal plate, in line with previous results [42], and to be massively repressed by *Phox2b*. *Sfrp1 *overexpression in the chicken spinal cord up-regulated the interneuron marker Lhx1,5. When co-expressed with *Sfrp1*, *Phox2b *was still capable of inducing Islet1,2, but became unable to repress expression of Lhx1,5. Hence, co-expression of *Phox2b *and *Sfrp1 *results in a mixed phenotype that is not normally encountered in the spinal cord, as also indicated by the observation that *Sfrp1 *partially restored the ability of *Phox2b*-transfected neurons to grow commissural axons. However, expression of *Pax2*, another interneuronal marker that was down-regulated by *Phox2b*, was already reduced by expression of *Sfrp1 *alone and continued to be down-regulated when Sfrp1 was co-expressed with *Phox2b*.

The Sfrps have been mostly described as secreted inhibitors of Wnt signaling, but there is evidence that they are able to promote rather than to inhibit Wnt signaling in some contexts and that they can act through Wnt-independent mechanisms [20-22]. In the *Wnt1*^-/-^; *Wnt3a*^-/- ^embryonic spinal cord, the dI4 (Lhx1,5^+^; Pax2^+^) subclass of interneurons has been found to be expanded at the expense of the dI1 and dI2 interneuron populations [43]. In line with these results, we found that *Sfrp1 *increased the population of Lhx1,5-expressing cells. *Sfrp1*, however, down-regulated *Pax2*, suggesting that the effect of *Sfrp1 *overexpression cannot be explained solely by inhibition of Wnt signaling. Gain of function experiments in the chicken retina have shown that *Sfrp1 *promotes neuronal differentiation and the generation of retinal ganglion cells and cones at the expense of other cell types by a mechanism that does not involve inhibition of Wnt signaling [44]. In our study, *Sfrp1 *overexpression in the spinal cord did not cause an obvious increase in generic neuronal differentiation. Still, our results suggest that Sfrp1 plays a role in neuronal subtype specification as has been observed in the retina.

Transcription factors of the B-, C- and E-group Sox proteins have been found to play key roles during early neural development [33,45-47]. By contrast, much less is known about the expression of the D-group Sox proteins in neural tissues and virtually nothing is known about their function during central nervous system development. In our screen, the D-group *Sox13 *gene was the only *Sox *gene that scored as a *Phox2b*-regulated gene. A previous study reported Sox13 to be expressed mainly by differentiating neurons in the ML [48]. At the slightly earlier stages and more caudal regions studied here, we found, by both ISH and antibody staining, Sox13 to be expressed throughout and confined to the VZ in both the mouse and chicken nervous systems. We found *Sox13 *to be expressed in the VZ in a salt-and-pepper pattern, patches of high signal intensity alternating with cells expressing lower levels, suggesting a highly dynamic expression pattern perhaps in relation to the commitment to differentiation. VZ cells that expressed constitutively high levels of *Sox13 *after transfection were proliferating as indicated by BrdU incorporation and many expressed the neural cell stem marker Sox2, but none the neuronal marker NeuN, whose expression in the ML was inhibited. These results may be interpreted to mean that the cells normally expressing lower levels were those destined to become neurons. In E10.5 ventral r4, Sox13 mRNA and protein expression, which was strong in more dorsal regions, was much weaker in the fbm progenitor domain, but invaded the normally *Phoxb*^+ ^region in the mutants. While Phox2b expression in the VZ and motor neuron production is prolonged in ventral r4, peak motor neuron production in r2 occurs at E9.5 and ceases around E10.5 together with Phox2b expression in the VZ, where the progenitors switch to the production of serotonergic neurons [1]. Consequently, in E9.5 ventral r2, *Sox13 *expression was absent from ventral progenitors, but became detectable there one day later. At this stage, the basic-helix-loop-helix factor Mash1 has been found to be required in this domain as both the sole provider of proneural activity and a determinant of the serotonergic phenotype [7]. As shown here, *Sox13 *expression did not expand into the ventral progenitor domain when *Mash1 *was absent, although *Phox2b *expression had ceased. One possibility is that *Sox13 *expression requires activation of the Notch pathway by its ligands, whose expression depends on proneural activity provided by Mash1, but more work is required to ascertain this. The idea that Sox13 may function as a Notch effector is also supported by the observation that its overexpression inhibits neurogenesis.

In contrast to what might have been expected from the pronounced down-regulation of *Sox13 *in *Phox2b*-expressing progenitors, *Phox2b *misexpression in spinal regions of the chicken neural tube did not result in downright repression, but areas of down-regulation alternated with regions with enhanced expression. Again, one reason may be that *Sox13 *expression depends on Notch activity, which may be repressed by *Phox2b *but enhanced in neighboring cells by the propensity of *Phox2b *to induce the Notch ligand Dll1 [8]. Constitutively high expression of *Sox13*, however, counteracted *Phox2b *activity in our ectopic expression paradigm. A major effect was to block the relocation of the transfected cells to the ML. In the presence of forced expression of *Sox13*, *Phox2b *was still able to promote cell cycle exit and neuronal differentiation, but now the Phox2b^+^, NeuN^+ ^and BrdU^- ^cells normally located in the ML accumulated in the VZ. Many of the doubly transfected cells expressed Sox2, which was totally absent from cells transfected with *Phox2b *alone, and Islet1,2 induction by *Phox2b *was inhibited in them. Hence, *Sox13 *appears to counteract the capacity of *Phox2b *to silence *Sox2 *expression and to induce a bm phenotype. However, blocking migration to the ML may still be the primary effect, since persistent *Sox2 *expression and inhibition of Islet1,2 induction may be consequences of the former. So far, the only *in vivo *function of *Sox13 *that has been reported is the promotion of, and a requirement for, proper development of the gamma/delta T cell lineage [25]. This has been attributed to the inhibition of Wnt signaling by direct interaction with the Wnt effector TCF1. Given that this also holds true for Sox13 function in the nervous system, it is interesting that two inhibitors of Wnt signaling, Sfrp1 and Sox13, appeared in our screen. However, Sfrp1 and Sox13 act on different processes: Sox13 inhibits generic neuronal differentiation while Sfrp1 affects cell type specification. Repression of both may thus be a prerequisite for proper development of bm neurons. Sox13 knockout mice die at birth from unknown reasons [25], and this may well be because of defective neural development. Our results showing that *Sox13 *inhibits neurogenesis and blocks migration to the ML give a first glance of the possible *in vivo *functions of *Sox13 *in neural development, opening the way for future mechanistic studies.

## Materials and methods

### Mice and embryo dissection

Phox2b^LacZ/+ ^mice [49] were intercrossed and the hindbrains of E10.5 embryos were dissected out and incubated for 2 minutes at 37°C with 1 μg/μl fluorescein di-(beta-D-galactopyranoside) (Sigma-Aldrich, Lyon, France). The left and right beta-galactosidase expressing domains of ventral r4 with the floorplate in between were excised under a fluorescent binocular and stored at -20°C in RNALater solution (Ambion, Austin, TX, USA). The rest of the embryo was used for genotyping as described by Pattyn *et al*. [49]. *Mash1+/- *mice (obtained from François Guillemot) were intercrossed and the embryos genotyped as described [50].

### RNA extraction and amplification

Four r4 samples of the same genotype (either *Phox2b*^*LacZ*/+ ^or *Phox2b*^*LacZ*/*LacZ*^) were pooled together and RNA was extracted and amplified using the RNAqueous-Micro Kit (Ambion) and the Message Amp aRNA amplification kit (Ambion) according to the instructions of the manufacturer. The quantity and the quality of the amplified RNA were verified using Agilent RNA 6000 Nano Assay and Bioanalyzer 2100 (Agilent Technologies, Massy, France).

### cDNA microarray hybridization

Microarray hybridization was done using two cDNA collections, the NIA Mouse 15 k clone sets [51] and the NeuroDev clone sets (Ecole normale supérieure local collection), both spotted on Ultra GAPS II slides (Corning, Avon, France) using a BioRobotics MicroGrid microarrayer (Genomic Solutions, Huntingdon, UK) by the Transcriptome platform of ENS, Paris. Amplified RNA (2 μg) was retrotranscribed and labeled with dUTP-Cy5 or dUTP-Cy3 (Amersham, Piscataway, NJ, USA) using Superscript III Reverse Transcriptase (Invitrogen, Carlsbad, CA, USA) in the presence of 5 μg of random primers and appropriate buffer (Invitrogen). RNA was degraded by alkaline lysis and cDNA was purified using a QIAquick purification kit (Qiagen, Courtaboeuf, France). CDNA microarrays were pre-hybridized for 1 h at 42°C in 5 × SSC, 0.1% SDS, 1% bovine serum albumin, washed with distilled water and dried. They were hybridized overnight at 42°C in 25% formamide, 5 × SSC, 0.1% SDS with Cy5- and Cy3-labeled cDNA, washed in 1 × SSC, 0.2% SDS at 42°C, in 0.1 × SSC, 0.2% SDS and two times in 0.1 × SSC at room temperature and then spin-dried. Four biological replicas (independent dissections) for each microarray (15 k and NeuroDev) were done and each of these replicas was hybridized independently twice by inverting the labeling dyes (dye-swap). A total of 16 hybridizations where thus performed, 8 using NIA 15 k microarrays and 8 others using NeuroDev slides.

### Data acquisition and analysis

The hybridized microarrays were scanned using Genepix 4000B (Molecular Devices, Sunnyvale, CA, USA)) and the resulting image files analyzed by GenePix Pro 5.0 software (Axon). For each GenePix output file, two filters were applied, one to clear out spots and another one to discard saturating spots where the median foreground intensity was greater than 60,000 in one of the two channels. The resulting median foreground intensities were normalized, without background signal subtraction, for dye bias using a global Lowess correction [52]. An expression matrix for each type of microarray (eight experiments each) was created gathering all normalized ratio data (M values). After normalization, we applied on each matrix a hierarchical clustering algorithm [53] using TMeV software [54] to detect a possible bias introduced by sample heterogeneity. The cluster obtained showed a clear separation according to dye-swap between hybridizations indicative of homogenous samples. The two matrices obtained were processed using the GEPAS web server [55] to remove genes with more than 30% missing values. A KNN impute step was performed to calculate missing values for the remaining genes using the 15 nearest neighbor profiles. A further scaling step was performed by dividing the ratios for each gene in one experiment by the experimental standard deviation and multiplying each value by the mean of all experimental standard deviations. The ratios were displayed such that a positive ratio describes a gene whose expression is higher in *Phox2b*^*LacZ*/+ ^than in *Phox2b*^LacZ/LacZ ^embryos. SAM [56] was used to search for differentially expressed genes and to take advantage of our four biological replicates to calculate the SAM score equivalent to the T-statistic value and to estimate the associated false discovery rate. We fixed the false discovery rate at 5% using the 90th percentile of falsely called genes. We chose in addition a SAM score above 3.5 or below -3.5 (for genes down- or up-regulated in the mutants, respectively) as an arbitrary cut-off since smaller scores increased the number of genes whose expression changes could not be confirmed by ISH.

### Expression vectors and electroporation

We used *pCAGGS::Phox2b-IRES2-EGFP *[2], *pCAGGS::Gap43*-*IRES2-EGFP*, *pCAGGS::Sox13-IRES2-EGFP*, *pCAGGS::Sfrp1-IRES2-EGFP *or *pCAGGS::IRES2-EGFP *to express mouse *Phox2b*, mouse *Gap43*, human *SFRP1 *(the latter two subcloned from IMAGE clones) and human *Sox13 *(subcloned from Ultimate ORF Clone IOH44934, Invitrogen) together with *GFP *or *GFP *alone in the chicken neural tube driven by a composite chicken beta actin-CMV promoter. The spinal region of HH12-14 embryonic chick neural tubes were electroporated *in ovo *with the appropriate expression vectors (2 mg/ml or 1 mg/ml) essentially as described [8]. Correct expression of all constructs was verified by ISH with the cognate probes. After electroporation, embryos were allowed to develop at 38°C for 24–72 h.

### Histological methods

Mouse embryos and well-electroporated chicken embryos, as assessed by GFP fluorescence, were dissected out, fixed in 4% paraformaldehyde overnight, embedded in Tissue-Tek^® ^OCT™ (Sakura-Finetek, Zoeterwoude, The Netherlands) (mouse embryos) or gelatin (chicken embryos) and analyzed on 12 or 20 μm transverse cryosections, respectively. The methods used for ISH immunohistochemistry were as described previously [2,8]. The following antibodies were used: rabbit anti-axonin-1 [27], mouse monoclonal anti-BrdU (1/100; Sigma), mouse monoclonal (Roche, Basel, Switzerland) and rabbit (Chemicon, Temecula, CA, USA) anti-GFP (1/400), mouse anti-Islet1,2 (1/100) [57], rabbit anti-Islet1 (1/500; Abcam, Paris, France), mouse anti-Lhx1,5 (Developmental Studies Hybridoma Bank), mouse anti-NeuN (1/500), rabbit anti-Sox2 (1/1,000; Abcam), rabbit anti-Sox13 [48] and adequate fluorescent secondary antibodies. For NeuN and Sox2 staining, sections were pre-treated with sodium citrate pH 6, 0.05% Tween-20, and for BrdU staining, sections were pretreated in 2N HCl, 0.5% Triton X-100 for 20 minutes at room temperature and neutralized. Apoptotic cells were detected using the Apo Tag kit (Oncor, Gaithersburg, MD, USA)) according to the manufacturer's instructions.

## Abbreviations

bm: branchiomotor; BrdU: bromo-deoxyuridine; E: embryonic day; fbm: facial bm; GFP: green fluorescent protein; hae: hours after electroporation; HH: Hamburger and Hamilton stage; IGF: insulin-like growth factor; ISH: *in situ *hybridization; ML: mantle layer; r: rhombomere; SAM: significance analysis of microarray; VZ: ventricular zone.

## Competing interests

The authors declare that they have no competing interests.

## Authors' contributions

PP devised and performed experiments and analyzed data, MRH performed experiments, SLC analyzed data, SR performed experiments, VH provided valuable reagents, CG devised research, analyzed data and wrote the article. All authors read and approved the final manuscript.

## Supplementary Material

Additional file 1 Genes down-regulated in *Phox2b*^*LacZ*/*LacZ *^embryos. A list of the 51 genes that were found to be significantly down-regulated in the ventral r4 of *Phox2b*^*LacZ*/*LacZ *^embryos according to the criteria presented in Materials and methods (SAM score above 3.5). The expression of the genes written in red was tested by ISH in *Phox2b*^*LacZ*/+ ^and *Phox2b*^*LacZ*/*LacZ*^embryos. Click here for file

Additional file 2 Genes up-regulated in *Phox2b*^*LacZ*/*LacZ *^embryos. A list of the 23 genes that were found to be significantly up-regulated in the ventral r4 of *Phox2b*^*LacZ*/*LacZ *^embryos according to the criteria presented in Materials and methods (SAM score below -3.5). The expression of the genes written in red was tested by ISH in *Phox2b*^*LacZ*/+ ^and *Phox2b*^*LacZ*/*LacZ*^embryos. Click here for file
