# Supplementary material for: Identification of Phox2b-regulated genes by expression profiling of cranial motoneuron precursors
Source: Neural Dev. 2008 Jun 19;3:14. doi: 10.1186/1749-8104-3-14 (PMC2441621; doi:10.1186/1749-8104-3-14)
Supplement: Additional file 1 — Genes down-regulated in Phox2bLacZ/LacZ embryos. A list of the 51 genes that were found to be significantly down-regulated in the ventral r4 of Phox2bLacZ/LacZ embryos according to the criteria presented in Materials and methods (SAM score above 3.5). The expression of the genes written in red was tested by ISH in Phox2bLacZ/+ and Phox2bLacZ/LacZembryos. [file 1749-8104-3-14-S1.doc]

| **Genes downregulated in *Phox2b^LacZ/LacZ^*embryos** | | | |  |  |  |  | |
| --- | --- | --- | --- | --- | --- | --- | --- | --- |
| score | log2 ratio | clone name | description | |  |  |  | |
| 11,5475 | 1,82424 | H3101B07 | Brain neurofilament-L | | | | | |
| 9,91623 | 1,85625 | H3157F06 | Growth associated protein 43 (Gap43) | | | | | |
| 7,87096 | 0,410948 | H3010D02 | Melanoma X-actin (Actx), | | | | | |
| 7,58992 | 0,329433 | ndv0392 | Meox 2 |  | |  | |  |
| 7,04873 | 0,829363 | ndv0222 | Amyloid beta (A4) precursor protein (App) | | | | | |
| 6,49977 | 0,94175 | H3117B04 | RIKEN cDNA 9030425E11 (adipocyte adhesion molecule) | | | | | |
| 6,02696 | 0,717375 | H3109A05 | CD24a |  | |  | |  |
| 5,10354 | 0,783875 | H3057B12 | Tubulin, beta 3 (Tubb3) | | | | | |
| 5,00937 | 0,60625 | H3022D10 | 22 kDa neuronal tissue-enriched acidic protein homolog | | | | | |
| 4,91552 | 0,359625 | H3058F06 | hypothetical Rhodopsin-like GPCR superfamily containing protein | | | | | |
| 4,86288 | 0,449125 | H3012A02 | Actin, beta, cytoplasmic (Actb) | | | | | |
| 4,51453 | 1,13962 | H3042F07 | Synuclein, gamma (Sncg) | | | | | |
| 4,49284 | 0,456375 | H3120F12 | Dynein, cytoplasmic, intermediate chain 2 (Dncic2) | | | | | |
| 4,27825 | 0,85525 | H3102B07 | Zinc finger protein, subfamily 1A, 4 (Znfn1a4) | | | | | |
| 4,24024 | 0,341125 | H3153G07 | Vhlh-interacting deubiquitinating enzyme 1 (Vdu1-pending) | | | | | |
| 4,22443 | 0,494625 | H3085H04 | RIKEN cDNA 2010004P11 gene RNA binding motif protein 18 | | | | | |
| 4,17416 | 0,260089 | H3151D03 | Kinesin-like protein KIF1B (Kif1b) | | | | | |
| 4,16702 | 0,41 | H3006H05 | calmodulin 2 (Calm2) | | |  | |  |
| 4,15722 | 0,182243 | H3106F06 | UNC51.1 serine/threonine kinase (Unc51.1) | | | | | |
| 4,13568 | 0,411125 | H3054A03 | RIKEN cDNA 2610306D21 gene (2610306D21Rik) | | | | | |
| 4,13168 | 0,37725 | H3142E11 | RIKEN cDNA 5830467E07 gene (5830467E07Rik) | | | | | |
| 4,1203 | 0,218322 | H3049E12 | Myeloid cell leukemia sequence 1 (Mcl1) | | | | | |
| 4,09797 | 0,608 | H3129G09 | Ubiquitin carboxy-terminal hydrolase L1 (Uchl1) | | | | | |
| 4,089 | 0,170659 | H3014C04 | Low density lipoprotein receptor | | | | | |
| 4,06356 | 0,344875 | H3015B09 | Actin-related protein 3 homolog (yeast) (Actr3) | | | | | |
| 3,92242 | 0,2389 | H3138D07 | Voltage-dependent anion channel 2 (Vdac2) | | | | | |
| 3,91728 | 0,269875 | H3019H11 | ATPase-like vacuolar proton channel (Atpl) | | | | | |
| 3,91534 | 0,33025 | H3061E04 | Cysteine dioxygenase 1, cytosolic (Cdo1) | | | | | |
| 3,83991 | 0,40425 | H3150D11 | Diméthylargininedimethylaminohydrolase 1 | | | | | |
| 3,80449 | 0,3505 | H3010F08 | Glucosaminyl (N-acetyl) transferase 2 | | | | | |
| 3,78285 | 0,254 | H3009G03 | Inositol (myo)-1(or 4)-monophosphatase 1 (Impa1) | | | | | |
| 3,7577 | 0,328968 | H3109A04 | Receptor (calcitonin) activity modifying protein 2 (Ramp2) | | | | | |
| 3,7523 | 0,390125 | H3120F11 | ATPase family AAA domain containing4 (Atad4) | | | | | |
| 3,7451 | 0,121838 | H3059H05 | Cadherin 1 (Cdh1) | | | | | |
| 3,74378 | 0,237651 | ndv0060 | Smad5 | | | | |  |
| 3,72992 | 0,36525 | H3058C11 | Fox-1 homolog (C. elegans) | | | | |  |
| 3,72195 | 0,30379 | H3122H01 | Paxillin-like protein (Hic5) | | | | | |
| 3,71555 | 0,276047 | ndv0257 | Map2k1 |  | |  | |  |
| 3,67814 | 0,511375 | H3059F01 | Ubiquitin carboxy-terminal hydrolase L1 (Uchl1) | | | | | |
| 3,67419 | 0,30625 | H3056E01 | Actin related protein 2/3 complex, subunit 5 (165 kDa) (Arpc5) | | | | | |
| 3,63462 | 0,396375 | H3066H10 | Mitogen activated protein kinase 8 (Mapk8) | | | | | |
| 3,6098 | 0,238872 | H3123B05 | Histone deacetylase 2 (Hdac2) | | | | | |
| 3,57257 | 0,192594 | H3071A11 | RNA-binding protein isoform G3BP-2a (G3BP2) | | | | | |
| 3,56702 | 0,264 | H3138C12 | Activity-dependent neuroprotective protein (Adnp) | | | | | |
| 3,54869 | 0,288607 | ndv0175 | Chaperonin subunit 6a (Cct6a) | | | | | |
| 3,54751 | 0,213663 | H3141C09 | spermatid perinuclear RNA-binding protein (Spnr) | | | | | |
| 3,54654 | 0,180933 | ndv0383 | Brn-3.2 |  | |  | |  |
| 3,54379 | 0,316625 | H3119E09 | RIKEN cDNA 3110043O21 gene (3110043O21Rik) | | | | | |
| 3,54311 | 0,603875 | H3009H02 | RUN and FYVE domain containing (Rufy3) | | | | | |
| 3,53951 | 0,529375 | H3122C07 | Stathmin (phosphoprotein P19) (PP19) (oncoprotein18) (op18) | | | | | |
| 3,53522 | 0,179752 | H3088A05 | MEF2A | | | | | |
