# Supplementary material for: Identification of Phox2b-regulated genes by expression profiling of cranial motoneuron precursors
Source: Neural Dev. 2008 Jun 19;3:14. doi: 10.1186/1749-8104-3-14 (PMC2441621; doi:10.1186/1749-8104-3-14)
Supplement: Additional file 2 — Genes up-regulated in Phox2bLacZ/LacZ embryos. A list of the 23 genes that were found to be significantly up-regulated in the ventral r4 of Phox2bLacZ/LacZ embryos according to the criteria presented in Materials and methods (SAM score below -3.5). The expression of the genes written in red was tested by ISH in Phox2bLacZ/+ and Phox2bLacZ/LacZembryos. [file 1749-8104-3-14-S2.doc]

| **Genes upregulated in *Phox2b^LacZ/LacZ^* embryos** | | |  |
| --- | --- | --- | --- |
| score | log2 ratio | clone name | description |
| -12,3435 | -1,16887 | H3126F05 | Insulin-like growth factor binding protein 5 (Igfbp5) |
| -6,91163 | -0,545636 | ndv0025 | Sfrp1 |
| -5,70488 | -0,357594 | H3134F06 | Extracellular matrix protein 2 (Ecm2) |
| -5,45056 | -0,280279 | H3087E05 | Chromatin assembly factor 1, subunit A (p150) (Chaf1a) |
| -5,37461 | -0,536451 | ndv0316 | Hes1 |
| -4,95926 | -0,182307 | H3155A12 | FGF receptor 1 (Fgfr1) |
| -4,71041 | -0,200388 | H3026H03 | P1 protein (P1.m) |
| -4,51894 | -0,453032 | H3120D01 | MEK kinase 3 |
| -4,26356 | -0,33953 | H3147B01 | TYRO3 protein tyrosine kinase 3 (Tyro3) |
| -4,15531 | -0,222235 | H3146E01 | Replication protein A2 (Rpa2) |
| -3,96489 | -0,233217 | H3079E05 | DNA polymerase epsilon (Pole) |
| -3,95842 | -0,211846 | H3029B11 | High mobility group protein I (Hmgi) |
| -3,9453 | -0,180402 | H3126F11 | Milk fat globule-EGF factor 8 protein (Mfge8) |
| -3,82038 | -0,272744 | H3095H03 | Regulatory factor (trans-acting) 2 (Rfx2) |
| -3,81941 | -0,182677 | H3078F10 | Xrcc1 |
| -3,81312 | -0,246882 | H3120F06 | ephrin-B3 (EFNB3) |
| -3,78512 | -0,609 | H3027E05 | Uridine phosphorylase (Upp) |
| -3,68682 | -0,194935 | H3143C08 | p53-associated cellular protein PACT |
| -3,611 | -0,165959 | ndv0305 | Transcobalamin II (Tcn2) |
| -3,60031 | -0,4082 | H3120G12 | KIAA1392 protein |
| -3,59996 | -0,190203 | H3002F08 | SRY-box containing gene 13 (Sox13) |
| -3,59462 | -0,160533 | H3053B07 | Tyrosine hydroxylase (Th) |
| -3,52982 | -0,183282 | ndv0435 | Tcf3 |
